# Prevalence of postpartum anaemia and iron deficiency by serum ferritin, soluble transferrin receptor and total body iron, and associations with ethnicity and clinical factors: a Norwegian population-based cohort study

**DOI:** 10.1017/jns.2022.45

**Published:** 2022-06-13

**Authors:** Marthe-Lise Næss-Andresen, Anne Karen Jenum, Jens Petter Berg, Ragnhild Sørum Falk, Line Sletner

**Affiliations:** 1 Department of General Practice, Institute of Health and Society, University of Oslo, Oslo, Norway; 2 General Practice Research Unit, Department of General Practice, Institute of Health and Society, University of Oslo, Oslo, Norway; 3 Department of Medical Biochemistry, Institute of Clinical Medicine, University of Oslo, Oslo, Norway; 4 Oslo Centre for Biostatistics and Epidemiology, Oslo University Hospital, Oslo, Norway; 5 Department of Paediatric and Adolescents Medicine, Akershus University Hospital, Lørenskog, Norway; 6 Institute of Clinical Medicine, University of Oslo, Oslo, Norway

**Keywords:** Anaemia, Cohort, Ethnic minorities, Iron deficiency, Postpartum iron status

## Abstract

Worldwide, there are limited data on the prevalence of postpartum anaemia and iron status. The aims of the present study were to assess the prevalence of anaemia and iron deficiency (ID) by three iron indicators 14 weeks postpartum, their relations to haemoglobin (Hb) and associations with ethnicity and clinical factors in a multi-ethnic population. We conducted a population-based cohort study of 573 women followed from early pregnancy. The prevalence of postpartum anaemia (Hb <12·0 g/dl) was 25 %. ID prevalence varied from 39 % by serum ferritin (SF <15 μg/l), to 19 % by soluble transferrin receptor (sTfR >4·4 mg/l) and 22 % by total body iron (TBI < 0 mg/kg). The mean Hb concentration was 12·8 g/dl in women with no ID, 12·6 g/dl in those with ID by SF only and 11·6 g/dl in those with ID by SF, sTfR and TBI. ID by sTfR and TBI defined by the current threshold values probably identified a more severe iron-deficient population compared with ID assessed by SF. Compared with Western Europeans, the prevalence of anaemia was at least the double in ethnic minorities (26–40 % *v*. 14 %; *P* < 0·01–0·05), and the prevalence of ID by sTfR and TBI, but not of ID by SF < 15 μg/l, was significantly higher in some minority groups. After adjustment for covariates, only South Asians had lower Hb and higher sTfR concentration. Insufficient iron intake, gestational anaemia or ID, and postpartum haemorrhage were associated with lower postpartum Hb concentration and poorer iron status.

## Introduction

The prevalence of postpartum anaemia in high-income countries is estimated to 10–30 %, but is generally higher in low- and middle-income countries^(1)^. Iron deficiency (ID) is considered the main cause of anaemia, due to bleeding during childbirth or inadequate dietary iron intake/uptake^(2–4)^. Women who have emigrated from low- and middle-income countries to Europe are considered a vulnerable group concerning several nutritional insufficiencies in pregnancy, e.g. ID, vitamin D deficiency and low use of folic acid^(5–9)^, but their postpartum iron status has scarcely been assessed.

ID occurs through a gradually reduction of iron stores, from being replete to being depleted and eventually absent, which consequently results in ID anaemia. ID can be measured by a variety of biomarkers. Serum ferritin (SF) concentration <15 or <12 μg/ml is widely used in the diagnosis of ID, and is considered a sensitive indicator of body iron stores in the absence of pregnancy, infection and inflammatory processes. The SF concentration below the threshold reflects depleted body iron stores, but cannot determine the severity of the ID^(10)^. Soluble transferrin receptor (sTfR) is an alternative iron marker suggested used when SF interpretations are hampered, and better reflects the cellular iron demand. In contrast to SF, sTfR is increased in ID. Elevated sTfR concentration reflects increasing cellular iron demand and falling Hb-synthesis, and the circulating sTfR and cellular iron demand are found to be proportional, thus reflecting early functional ID^(11)^. Together, SF and sTfR cover the full range of iron status, and combining them in a new model, called total body iron (TBI) is considered to better predict the absence of bone marrow iron^(10)^. While this marker is now widely used in the United States, it has not been implemented in Europe^(12)^. A positive value of TBI represents iron storage, while negative values indicate a deficient iron supply to peripheral tissues^(12,13)^.

We have previously shown that in early pregnancy ethnic minority women had more ID for all three iron indicators, also when adjusting for covariates^(5)^. Postpartum anaemia and ID may have adverse short – and long-term health implications for the mother and her child, such as fatigue, lower work capacity and increased risk of postpartum depression and poorer mother–child interaction^(4)^. Furthermore, maternal mortality increases with severe anaemia, as well as the risk of infections in the puerperium and poorer wound healing^(4)^.

There are limited data on the prevalence of postpartum anaemia and iron status. The aims of the present study were to assess the prevalence of anaemia and ID by three iron indicators 14 weeks after delivery, including the relations between the ID indicators and their relations to haemoglobin (Hb), and their associations with ethnicity and clinical factors.

## Subjects and methods

### Study population and data selection

Our data is from the STORK-Groruddalen study of multi-ethnic pregnancies in Oslo, Norway, collected at public Child Health Clinics for primary antenatal care in the period 2008–10 in three administrative districts in Oslo. The study methods have been described in detail elsewhere^(14)^. In short, information, material and questionnaires were translated into Arabic, English, Sorani, Somali, Tamil, Turkish, Urdu and Vietnamese and quality checked by bilingual health professionals. Pregnant women were eligible if they (I) lived in the district, (II) planned to give birth at one of the two study hospitals, (III) were in <20 gestational week (GW) (IV) were not suffering from diseases necessitating intensive hospital follow-up during pregnancy, (V) could communicate in Norwegian or any of the specified languages and (VI) were able to provide written informed consent.

In total, 823 pregnant healthy women from 65 countries were included in early pregnancy (mean GW 8–20), with planned follow-up visits in GW 28 and about 3 months after delivery. The time point for the postpartum visit was chosen to ensure a high attendance rate, as at this point, most women have recovered from birth, and have established daily routines. Furthermore, physiological pregnancy-related changes, including haemodilution, will have returned to normal. At all three study visits, questionnaire data were collected through interviews by authorised study personnel, assisted by professional interpreters when needed^(14)^. The questionnaire data covered a wide range of health issues, and clinical measurements were collected according to the study protocol. Participating women were found representative for the main ethnic groups of pregnant women attending the Child Health Clinics^(14)^. Ethical approval was obtained from The Regional Ethics Committee.

### Outcome measures

Postpartum anaemia was defined as Hb concentrations <12·0 g/dl^(3,15)^ measured 14 weeks post-delivery. In addition, we used three established definitions for ID; SF concentration <15 μg/l, the primary indicator used by the WHO^(16)^, sTfR concentration >4·4 mg/l according to the manufacturer's guidelines, and TBI<0 mg/kg^(13)^. SF concentration below the threshold indicates depleted body iron stores, while an increased sTfR concentration reflects early functional ID and TBI is meant to be a quantitative estimate of the iron status^(10–13,17)^. Iron-deficiency anaemia (IDA) was defined as anaemia in the presence of ID by any iron indicator.

### Measurements of iron indicators and anaemia

Blood samples were drawn at all visits^(5,14)^. Hb (g/dl) and SF (μg/l) were analysed consecutively at the Department of Multidisciplinary Laboratory Medicine and Medical Biochemistry at Akershus University Hospital, Oslo, Norway. Hb was measured using an SLS method (XE 5000 from Sysmex; inter-assay coefficient of variation (CV) 0·7 %). SF was measured using an electro-chemiluminescence immunoassay (ECLIA) method (Unicel DxI 800 from Beckman Coulter; inter-assay CV <7 %). Blood samples were frozen and biobanked at −80 °C. In 2016, sTfR and high sensitivity C-reactive protein (CRP) were analysed from biobanked serum samples at the Department of Medical Biochemistry at Oslo University Hospital, Oslo, Norway. CRP was measured by a particle-enhanced turbidimetric immunoassay (CRP Vario from Sentinel on Vitros 5.1 FS; inter-assay CV <5 %), and sTfR by ELISA (Modular P800 from Roche; inter-assay CV <5 %^(18)^). We calculated TBI according to Cook^(13)^ from the ratio of sTfR concentration (by Flowers assay) to SF concentration: −[log_10_ (sTfR × 1000 ÷ SF) − 2·8229] ÷ 0·1207). To convert our Roche sTfR concentration to Flowers sTfR concentrations, we used the conversion equation Flowers sTfR 1·5 × Roche sTfR + 0·35 mg/l^(13)^.

### Socio-demographic variables

Ethnicity was defined as each participant's country of birth, or the participant's mother's country of birth if the participants’ mother was born outside Europe or North America^(14,19)^, and grouped as Western Europeans (Norway, other Western European countries and North America), South Asians (primarily Pakistan and Sri Lanka), Middle Easterners (primarily Iraq, Morocco and Turkey), East Asians (primarily Vietnam and The Philippines), Sub-Saharan Africans (primarily Somalia) and Eastern Europeans (primarily Poland, Kosovo and Russia). Maternal age was calculated from date of birth and date at enrolment in the present study. Parity was dichotomised into primiparous (first pregnancy lasting >22 weeks) and parous (one or more previous births) women. Pre-pregnancy body mass index (BMI, kg/m^2^) was calculated from self-reported weight before pregnancy and height measured at inclusion^(14)^. Gestational age was primarily derived from the first day of the mother's last menstrual period (LMP), but ultrasound-derived gestational age was used in 7 % of pregnancies where there were reasons to believe that the LMP-derived GA was uncertain^(20)^.

Most variables reflecting socioeconomic position (SEP) and level of integration are strongly correlated, although representing different dimensions of societal and contextual factors. Individual and household markers of maternal present SEP and variables related to the level of integration were therefore entered into a principal components analysis (PCA). Two separate, uncorrelated components were extracted^(19)^. The first component was strongly correlated with predefined markers reflecting integration such as language skills, time of residence, social interaction with ethnic Norwegians and use of Norwegian media, and was skewed to the right, as all ethnic Norwegians had high scores. Due to the skewed distribution, and as we were mainly interested in the potential effect of low integration, and to ease the interpretation of our results, the integration score was further dichotomised as the 40 % with the lowest scores *v*. the 60 % with highest scores. This pragmatic cut-point had a sufficient number in the lowest category and still carried important information. The second component had strong correlations with the predefined individual and household markers of SEP such as educational level, occupational class, employment status, renting tenure and rooms per person in the household, and was normally distributed, with a higher score reflecting higher SEP. Maternal early life SEP was derived from a separate PCA of three childhood socio-demographic variables (family occupational class (highest of mother and father), rooms per person in household and family ownership of car, all referring to maternal age of 10 years), and was also normally distributed. Time of residence was calculated from the participants’ self-reported year of arrival to Norway, and the participants’ Norwegian language skills were categorised as good and poor, derived from the their need of interpreter at the study visits^(19)^.

### Variables potentially associated with iron metabolism

Of ethical reasons, all participants with Hb <10·0 g/dl during pregnancy were informed by letter, and encouraged to seek their family doctor. Women with SF <20 μg/l and Hb >10 g/dl were recommended to use 30–50 mg iron supplementation per day. All participants were asked about their intake of iron supplements during the past 2 weeks at all three visits. Iron supplementation was dichotomised into ‘yes’, covering daily or intermittent iron supplements, and ‘no’. Data from a food frequency questionnaire, developed to capture dietary patterns in a multi-ethnic sample, were collected in GW 28. Four clusters were extracted using the Ward's method. Clusters were referred to as ‘a healthier dietary pattern’ *v*. three ‘less healthy dietary patterns’^(21)^, here dichotomised into ‘healthy’ and ‘unhealthy’. The ‘healthy dietary pattern’ represented more frequent intake of fruit, vegetables, wholegrain bread with pate and meat spread, and meat, i.e. food items representing relatively high intake of iron. From questions about their medical history, we categorised three groups: (1) no medical conditions associated with anaemia or ID, (2) self-reported chronic illness or medication associated with ID or normochromic anaemia (i.e. kidney or rheumatic disease, use of carbamazepine or infliximab) and (3) self-reported chronic illness or medication associated with ID or hypochromic anaemia (i.e. gastrointestinal disease or Copper IUD use before conception (associated with heavier menstrual bleeding and a poor iron status prior to their pregnancy))^(5)^. Haemoglobinopathy was either self-reported, identified from the HPLC (Tosoh G8, Tosoh Corporation) analysis of HbA1c (glycated Hb) or based on microcytic anaemia in the absence of low SF.

### Birth-related variables potentially associated with postpartum iron status

We have detailed data on birth complications extracted from hospital birth records. We categorised delivery mode into (1) normal vaginal delivery, (2) instrumental vaginal delivery (i.e. forceps or vacuum-assisted vaginal delivery), (3) elective caesarean section and (4) emergency caesarean section. Postpartum blood loss after delivery was extracted from the hospital's birth record (mainly reflecting blood loss directly related to birth); and further dichotomised into <500 ml and ≥500 ml – the last category defined as postpartum haemorrhage. Due to small numbers of each type of birth complications, we also constructed a composite variable reflecting the presence of at least one of the following complications; episiotomy, third- or fourth-degree perineal tear, obstructed labour and manual removal of placenta as an outcome.

### Sample size

Of the 823 (74 % of invited participants) women included in early pregnancy, 644 (78 %) attended at the postpartum visit in mean postpartum week 13·9 (sd ± 2·5) (flowchart, Supplementary Figure S1). For the present study, we included participants with no missing values for SF, sTfR and TBI at the postpartum visit, resulting in a total sample of 573 women (89 % of those attending the postpartum visit). There were no significant differences between the study sample and the 250 excluded women regarding age, parity, pre-pregnant BMI and SEP (data not shown). However, the study sample consisted of a slightly larger proportion of ethnic minority women as they were prioritised for fasting blood samples at the postpartum visit due to resource limitations, compared with the excluded women^(22)^.

### Statistical analyses

**Fig. 1.**
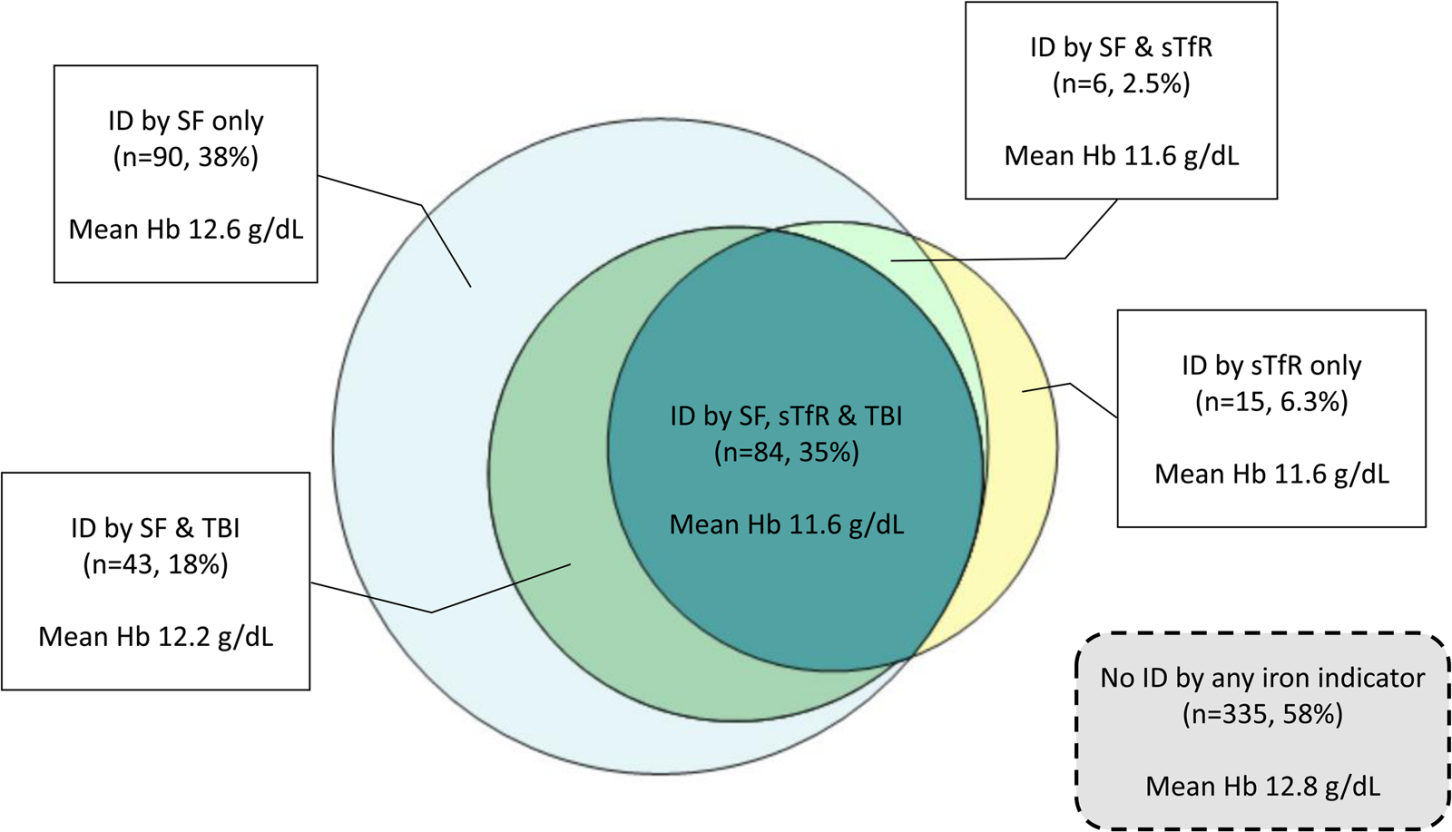
Venn diagram for postpartum women with iron deficiency by ≥1 of the three iron indicators serum ferritin, soluble transferrin receptor and total body iron (*n* 238) 14 weeks postpartum in the STORK-Groruddalen study^a^. Hb, haemoglobin; ID by SF, iron deficiency by serum ferritin concentration <15 μg/l; ID by sTfR, iron deficiency by soluble transferrin receptor concentration >4·4 mg/l; ID by TBI, iron deficiency by total body iron concentration <0 mg/kg. ^a^The STORK-Groruddalen multi-ethnic pregnancy cohort from Oslo, Norway, 2008–10.

**Fig. 2.**
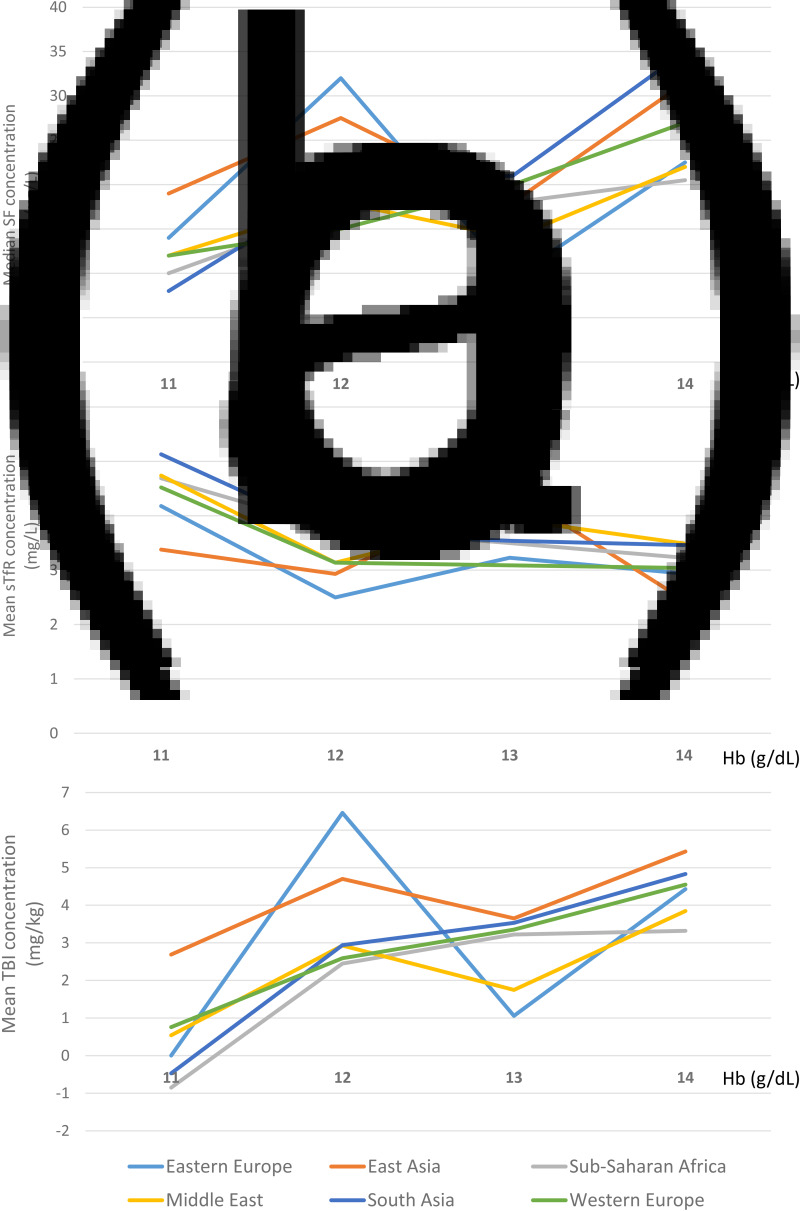
Median serum ferritin concentration (μg/l), mean soluble transferrin receptor concentration (mg/l) and mean total body iron concentration (mg/kg) in four haemoglobin concentration intervals (g/dl)^a^ at the postpartum visit in the STORK-Groruddalen multi-ethnic pregnancy cohort from Oslo, Norway, 2008–10. Hb, haemoglobin; SF, serum ferritin; sTfR, soluble transferrin receptor; TBI, total body iron. ^a^Haemoglobin as grouped midpoint; 11 (8·0–11·9); 12 (12·0–12·5); 13 (12·6–13·0) and 14 (13·1–15·0).

The STORK-Groruddalen study was originally designed to identify ethnic differences in the prevalence of gestational diabetes and aimed at including 800 women. In the present study, we take advantages of the collected date and performed regression models based on the large sample size available. Descriptive statistics are presented as frequencies with proportions for categorical variables and mean with standard deviations (sd) or medians with interquartile range for continuous variables. The sTfR, TBI and Hb values were approximately normally distributed (Table 1). We calculated percentages of abnormal values for SF (<15 μg/l), sTfR (>4·4 mg/l), TBI (<0 mg/kg) and Hb (<12·0 g/dl) for the total sample, and for each ethnic group. The differences in prevalence between Western Europeans and each non-Western group were tested by *χ*^2^ tests (Table 2). We used a scaled Venn diagram to illustrate the degree of overlap between measures of ID, and further to illustrate their relations to Hb by measuring mean Hb concentration in the groups with ID defined by the different iron indicators (Fig. 1). We also categorised Hb concentration into four groups, presented at the group midpoint, to explore the distribution of the three different iron indicators by ethnicity (Fig. 2). Furthermore, we performed sensitivity analysis, using the threshold of SF <12 μg/l for the prevalence of ID to ease comparison to other studies using this threshold and to the prevalence rates between the different iron indicators (Supplementary Table S1).

**Table 1.** Socio-demographic characteristics of the total sample in the STORK-Groruddalen study stratified into Western Europeans and non-Western women, and further into ethnic minority groups^a^

		Total		Western Europe		Non-Western		South Asia		Middle East		Sub-Saharan Africa		East Asia		Eastern Europe	
		*n* or means	sd or %	*n* or means	sd or %	*n* or means	sd or %	*n* or means	sd or %	*n* or means	sd or %	*n* or means	sd or %	*n* or means	sd or %	*n* or means	sd or %
	*n*	573	100	217	38	356	62	157	27	94	16	38	7	33	6	34	6
Postpartum week	566	13·9	2·5	13·6	2·2	14·1	2·5	14·0	2·5	14·0	2·7	14·4	2·2	14·7	3·3	13·7	1·9
Age at inclusion, years	573	29·7	4·8	30·9	4·6	29·2	4·8	28·7	4·4	29·7	5·6	29·0	5·1	30·9	4·4	28·4	4·3
Parity	564																
Primiparous		260	46	113	53	147	42	65	42	30	32	17	47	13	39	22	65
Multiparous		304	54	99	47	205	58	91	58	63	68	19	53	20	61	12	35
Pre-pregnant Body Mass Index, kg/m^2^	564	24·6	4·8	25·0	4·7	24·2	4·5	23·8	4·1	25·5	2·9	25·0	4·7	22·0	3·2	23·9	4·8
Maternal adult socioeconomic position^b^	570	0·01	1·0	0·5	0·8	−0·3	0·9	−0·2	0·8	−0·5	0·9	−1·0	1·2	0·1	0·7	0·1	1·1
Low social integration^c^	573	239	42	12	5·5	227	64	100	64	62	66	20	53	20	61	25	74
Early life socioeconomic position^d^	565	− 0·01	1·0	0·8	0·7	−0·5	0·8	−0·5	0·8	−0·5	0·8	−0·4	0·9	−0·6	0·9	−0·3	0·8
Gestational iron deficiency or anaemia^e^																	
Gestational ID by SF	572	178	31	26	12	152	43	75	48	37	39	20	54	9	27	11	32
Gestational ID by sTfR	562	32	6	0	0	32	9	19	12	9	10	4	12	0	0	0	0
Gestational ID by TBI	562	63	11	1	1	62	18	33	21	15	16	9	27	2	6	3	9
Gestational anaemia	564	32	6	3	1	29	8	17	11	6	7	4	11	2	6	0	0
Self-reported iron supplement use^f^																	
In early pregnancy	564	101	18	25	12	76	22	38	24	16	17	9	25	10	30	3	9
In gestational week 28	539	232	43	65	32	167	50	84	56	38	44	18	51	11	34	16	50
Postpartum	555	124	22	46	22	78	23	40	27	20	22	6	16	4	13	8	24
Dietary pattern^g^	555																
Healthy		164	30	122	58	42	12	14	9	6	7	5	15	7	22	10	29
Unhealthy		391	70	89	42	302	88	140	91	84	93	29	85	25	78	24	71
Chronic illness/medication	564																
Yes, associated with normochromic anaemia^h^		14	3	5	2	9	3	2	1	4	4	2	6	1	3	0	0
Yes, associated with hypochromic anaemia^i^		55	10	25	12	30	9	13	8	7	8	2	6	5	15	3	9
Delivery mode	573																
Normal vaginal delivery		473	83	175	81	298	84	134	85	79	86	30	79	27	82	28	82
Instrumental vaginal delivery^j^		58	10	21	10	37	10	17	11	8	9	6	16	3	9	3	9
Elective Caesarean section		29	5	16	7	13	4	5	3	1	1	1	3	3	9	3	9
Emergency Caesarean section		68	12	25	12	43	12	18	12	12	13	7	18	3	9	3	9
Birth complications	573																
Episiotomy		65	11	27	13	38	11	15	10	11	12	3	8	4	12	5	15
Third- or fourth-degree perineal tear		15	3	7	3	8	2	4	3	0	0	2	5	2	6	0	0
Obstructed labour		33	6	15	7	18	5	5	3	6	6	1	3	1	3	5	15
Manual removal of placenta		25	4	13	6	12	3	3	2	8	9	1	3	0	0	0	0
Postpartum haemorrhage^k^		33	6	15	7	18	5	5	3	6	6	1	6	1	3	5	15

Hb, haemoglobin; ID, iron deficiency; SEP, socioeconomic position; SF, serum ferritin; sTfR, soluble transferrin receptor; TBI; total body iron.

a The STORK-Groruddalen multi-ethnic pregnancy cohort from Oslo, Norway, 2008–10.

b Variable derived from a principal components analysis of predefined individual and household markers SEP, with a higher score reflect higher SEP.

c Variable derived from a principal components analysis of predefined markers reflecting integration such as language skills, time of residence, social interaction with ethnic Norwegians and use of Norwegian media, with a higher score reflect higher social integration. ‘Low social integration’ represents participants belonging to the 40 % with the lowest scores.

d Variable derived from a principal components analysis of three childhood socio-demographic variables representing maternal SEP at age 10 years, with a higher score reflecting higher SEP.

e Gestational iron deficiency by (1) SF <15 μg/l; (2) sTfR >4·4 mg/l or (3) TBI <0 mg/kg; and gestational anaemia by trimester-specific haemoglobin <10·5 or 11·0 g/dl, analysed in mean gestational week 15·1.

f Self-reported intake of iron supplements during the past 2 weeks at all three study visits dichotomised into ‘yes’, covering daily or intermittent iron supplements, and ‘no’.

g Data from a food frequency questionnaires collected in GW 28; four clusters were extracted using the Ward's method. Clusters were referred to as ‘a healthier dietary pattern’ *v*. three ‘less healthy dietary patterns’; here dichotomised into ‘healthy’ and ‘unhealthy’ dietary pattern.

h Self-reported chronic illness or medication associated with normochromic anaemia (i.e. kidney or rheumatic disease, use of carbamazepine or infliximab).

i Self-reported chronic illness or medication associated with ID and hypochromic anaemia (i.e. gastrointestinal disease or Copper intrauterine device use before conception).

j Assisted vaginal delivery through forceps or vacuum.

k Excessive blood loss (≥500 ml) after delivery.

**Table 2.** Values for serum ferritin, soluble transferrin receptor (sTfR), total body iron (calculated from ferritin and sTfR concentrations) and haemoglobin concentration, and prevalence of abnormal values (iron deficiency and anaemia) 14 weeks postpartum in the STORK-Groruddalen study^a^

	*n*	SF, μg/l	sd or IQR	sTfR, mg/l	sd or IQR	TBI, mg/kg	sd or IQR	Hb, g/dl	sd or IQR
Mean in total sample^a^	573	23	18	3·7	1·7	2·7	3·7	12·5	1·0
Median in total sample^a^	573	18	10, 32	3·3	2·7, 4·1	3·1	0·4, 5·4	12·6	11·9, 13·2
Prevalence of abnormal values^b^^,^^c^	*n*	%	95 % CI	%	95 % CI	%	95 % CI	%	95 % CI
Total sample^a^	573	39	35, 43	19	16, 22	22	19, 26	25	22, 29
Ethnic group^d^									
Western Europe	217	35	29, 42	12	8, 17	15	11, 20	14	10, 19
South Asia	157	44	36, 52	30	23, 38**	30	23, 37**	40	33, 48**
Middle East	94	39	30, 50	22	15, 32*	29	20, 39**	27	19, 37**
Sub-Saharan Africa	38	50	34, 66	21	11, 38	32	18, 49**	26	14, 43*
East Asia	33	36	21, 55	12	4, 29	15	6, 33	33	19, 52**
Eastern Europe	34	32	8, 50	6	1, 22	18	8, 35	18	8, 36

Hb, haemoglobin; ID, iron deficiency; SF, serum ferritin; sTfR, soluble transferrin receptor; TBI; total body iron.

a The STORK-Groruddalen multi-ethnic pregnancy cohort from Oslo, Norway, 2008–10. *N* 573 for serum ferritin, *n* 568 for soluble transferrin receptor (sTfR) and total body iron and *n* 569 for haemoglobin.

b Abnormal values are presented as percentage (95 % CI), defined as serum ferritin <15 μg/l, soluble transferrin receptor (sTfR) >4·4 mg/l, total body iron <0 mg/kg and haemoglobin <12·0 g/dl.

c Haemoglobinopathy (*n* 4) was either self-reported, identified from the HPLC (Tosoh G8, Tosoh Corporation) analysis of glycated haemoglobin, or from a combination of microcytic anaemia and high ferritin.

d The difference in the prevalence of abnormal values between Western Europeans and each non-Western group were tested by *χ*^2^ test.

*P < 0·05, **P < 0·01.

To examine associations between ethnicity, maternal factors before and during pregnancy, birth complications, and postpartum anaemia and ID, we performed linear regression analyses with Hb, sTfR and TBI as continuous outcome variables, and logistic regression analyses with SF <15 μg/l as a dichotomous outcome variable due to its skewed distribution. Factors of particular clinical relevance, such as ethnicity, gestational anaemia and ID, postpartum haemorrhage, parity, dietary pattern and iron supplementation were hence forced into the models. However, other potentially relevant factors with *P*-value <0·2 in the univariate analysis were also included into the multiple regression analyses, but only included in the final model if still significantly associated with the outcome after a stepwise backward elimination process (Table 3). Interactions with ethnicity were examined graphically and by entering cross-product terms, one-by-one, into the model. We *a priori* defined an interaction to be significant if the *P*-value was <0·01 and consistent for Hb and all three iron indicators. No significant interactions were observed.

**Table 3.** Logistic regression analysis of serum ferritin <15 μg/l, and linear regression analyses of soluble transferrin receptor, total body iron and haemoglobin concentration 14 weeks postpartum in the STORK-Groruddalen study^a^

	SF <15 μg/dl				sTfR, mg/l				TBI, mg/kg				Hb, g/dl			
	OR	95 % CI	adj OR	95 % CI	*β*	95 % CI	adj *β*	95 % CI	*β*	95 % CI	adj *β*	95 % CI	*β*	95 % CI	adj *β*	95 % CI
	
		*R*^2^ 0·13				*R*^2^ 0·14				*R*^2^ 0·15				*R*^2^ 0·17	
Ethnicity (Western European = reference)																
South Asia	1·4	0·9, 2·2	1·1	0·6, 1·7	0·9	0·6, 1·3**	0·8	0·4, 1·2**	−1·2	−2·0, −0·05**	−0·4	−1·2, 0·5	−0·6	−0·8, −0·4**	−0·4	−0·7, −0·2**
Middle East	1·2	0·7, 1·9	0·8	0·4, 1·5	0·6	0·1, 1·0**	0·4	−0·1, 0·9	−0·9	−1·8, −0·01*	−0·1	−1·1, 0·9	−0·2	−0·8, 0·01	0·03	−0·3, 0·3
Sub-Saharan Africa	1·8	0·9, 3·6	1·0	0·4, 2·3	0·5	−0·1, 1·1	0·4	−0·3, 1·0	−1·2	−2·5, 0·1	−0·1	−1·5, 1·3	−0·4	−0·8, −0·03*	−0·1	−0·5, 0·3
East Asia	1·0	0·5, 2·2	0·8	0·3, 1·8	0·2	−0·4, 0·8	0·2	−0·4, 0·9	0·6	−0·7, 1·9	0·9	−0·5, 2·2	−0·4	−0·8, −0·1*	−0·2	−0·6, 0·2
East Europe	0·9	0·4, 1·9	0·5	0·2, 1·3	−0·1	−0·7, 0·5	−0·1	−0·7, 0·5	0·01	−1·3, 1·3	0·8	−0·5, 2·2	0·3	−0·02, 0·7	0·5	0·1, 0·9*
Postpartum week	1·0	0·9, 1·0			−0·1	−0·1, −0·01*	−0·3	−0·1, −0·03**	0·1	−0·04, 0·2			0·02	−0·01, 0·05		
Age, per 5 year	0·8	0·7, 1·0*			−0·2	−0·4, −0·1**			0·7	0·4, 1·0**	0·5	0·2, 0·9**	0·1	−0·01, 0·02	0·1	0·01, 0·2*
Multiparous (primiparous = reference)	0·6	0·4, 0·8**	0·6	0·4, 0·8**	−0·2	−0·5, 0·1	−0·3	−0·6, −0·04*	−0·1	−0·4, 0·2	0·8	0·1, 1·4*	−0·03	−0·1, 0·1	−0·2	−0·3, 0·02
Pre-pregnant Body Mass Index, per 5 kg/m^2^	1·0	0·8, 1·2			0·2	0·03, 0·3*			−0,1	−0·4, 0·4			0·03	−0·1, 0·1		
Adult socioeconomic position^b^	0·8	0·7, 0·9*			−0·3	−0·4, 0·1**			0·6	0·3, 0·9**			0·2	0·1, 0·2**		
Early life socioeconomic position^c^	0·9	0·7, 1·0			−0·2	−0·4, −0·1**			0·3	0·01, 0·6*			0·2	0·1, 0·3**	0·1	0·03, 0·2*
Gestational ID or anaemia (no = reference)^d^	1·3	0·9, 1·8	1·6	1·0, 2·5*	2·0	1·4, 2·6**	1·7	1·1, 2·4**	−2·5	−3·4, −1·5**	−2·5	−3·5, −1·5**	−0·9	−1·3, −0·6**	−0·9	−1·3, −0·6**
Iron supplementation use in GW 28 (no = reference)^e^	0·6	0·4, 0·9*	0·5	0·3, 0·7**	−0·3	−0·6, −0·004*	−0·6	−0·9, −0·3**	0·8	0·1, 1·4*	1·3	0·7, 1·9**	−0·002	−0·2, 0·2	0·2	0·004, 0·3*
Unhealthy dietary pattern (healthy = reference)^f^	2·2	1·5, 3·3**	2·8	1·7, 4·5**	0·6	0·3, 1·0**	0·4	0·1, 0·8*	−1·5	−2·2, −0·8**	−1·3	−2·1, −0·5**	−0·2	−0·4, −0·1**	0·04	−0·2, 0·2
Chronic illness/medication associated with normochromic anaemia (no = reference)^g^	1·5	0·5, 4·4			0·8	−0·1, 1·7			−0·1	−2·0, 1·9			−0·8	−1·3, −0·2**	−0·8	−1·4, −0·3**
Chronic illness/medication associated with hypochromic anaemia (no = reference)^h^	0·7	0·4, 1·3			−0·2	−0·7, 0·3			0·4	−0·7, 1·4			−0·1	−0·3, 0·2	−0·1	−0·3, 0·2
Operative delivery (no = reference)^i^	1·3	0·9, 1·9			−0·03	−0·3, 0·3			−0·5	−1·2, 0·1			0·01	−0·2, 0·2		
Postpartum haemorrhage (<500 mL = reference)	2·9	1·4, 6·0**	3·3	1·5, 7·4**	0·3	−0·3, 0·9	0·3	−0·3, 0·9	−1·6	−2·9, −0·3*	−2·0	−3·3, −0·7**	−0·2	−0·5, 0·2	−0·4	−0·8, −0·1*
Birth complications (no = reference)^j^	1·4	0·9, 2·2			0·07	−0·3, 0·5			−0·8	−1·6, −0·04*			−0·1	−0·3, 0·2		

Adj, adjusted; ID, iron deficiency; GW, gestational week; Hb, haemoglobin; SEP, socioeconomic position; SF, serum ferritin; sTfR, soluble transferrin receptor; TBI; total body iron.

a The STORK-Groruddalen multi-ethnic pregnancy cohort from Oslo, Norway, 2008–10. Multivariable regression analyses with stepwise backward elimination; ethnicity and clinical relevant variables were forced into the model.

b Variable derived from a principal components analysis of predefined individual and household markers of SEP, with a higher score reflect higher SEP.

c Variable derived from a separate principal components analysis of three childhood socio-demographic variables representing maternal SEP at age 10 years, with a higher score reflecting higher SEP.

d Gestational iron deficiency by (1) SF <15 μg/l; (2) sTfR >4·4 mg/l or (3) TBI <0 mg/kg; and gestational anaemia by trimester-specific haemoglobin < 10·5 or 11·0 g/dl, analysed in mean gestational week 15·1.

e Self-reported intake of iron supplements during the past 2 weeks at all three study visits dichotomised into ‘yes’, covering daily or intermittent iron supplements, and ‘no’.

f Data from a food frequency questionnaires collected in GW 28; four clusters were extracted using the Ward's method. Clusters were referred to as ‘a healthier dietary pattern’ *v*. three ‘less healthy dietary patterns’; here dichotomised into ‘healthy’ and ‘unhealthy’ dietary pattern.

g Self-reported chronic illness or medication associated with normochromic anaemia (i.e. kidney or rheumatic disease, use of carbamazepine or infliximab).

h Self-reported chronic illness or medication associated with ID and hypochromic anaemia (i.e. gastrointestinal disease or Copper intrauterine device use before conception).

i Operative delivery: Caesarean section (elective and emergency) or assisted vaginal delivery (forceps or vacuum), with normal vaginal delivery as a reference.

j A composite variable created by combining following four birth complication; episiotomy, third- and fourth-degree perineal tear, obstructed labour and manual removal of placenta.

* *P* < 0·05, **P < 0·01.

As ethnicity is a broader concept than geographical ancestry, we also explored the impact of level of social integration, as alternative explanatory variables. First, we performed multivariable regression analyses replacing the ethnicity variable with the dichotomous variable low and high social integration (Supplementary Table S2). Second, we conducted sensitivity analyses in a sub-sample of ethnic minority women (*n* 332), dichotomised into ‘South Asian’ or ‘other’ ethnic origin, and explored if social integration could explain the differences observed between ethnic minority groups (Supplementary Table S3).

Results from linear regressions are presented as β-coefficients and results from logistic regression as odds ratios (ORs), both with accompanied 95 % confidence intervals (CIs). Model fit is presented by adjusted *R*^2^ or Nagelkerke *R*^2^, as appropriate. *P*-values <0·05 were considered statistically significant. SPSS version 25 and Stata version 15 were used for statistical analysis.

## Results

### Sample characteristics

Of the 573 women who constitute the sample 14 weeks postpartum sample, 62 % had ethnic origin outside Western Europe, mean age at inclusion was 29·7 (sd ± 4·8) years, mean pre-pregnant BMI was 24·6 (±4·8) kg/m^2^, and 46 % were primiparous (Table 1). Non-Western women were younger, and more often reported a dietary pattern categorised as less healthy compared with Western European women. They also had a lower socioeconomic position, both in childhood and as adults, represented by lower SEP-scores generated from the PCA analyses of several individual and household socio-demographic variables.

### Prevalence of anaemia and ID, relations between ID indicators and relations with anaemia

The mean Hb concentration was 12·5 ± 1·0 g/dl at the postpartum visit, and the overall prevalence of anaemia was 25 %, but the prevalence of ID differed by iron indicator, and was significantly higher by SF than by sTfR and TBI (Table 2). Only four women with haemoglobinopathy were identified. The Venn diagram (Fig. 1) illustrates that among the 298 women with ID by any indicator, 35 % had ID by all three indicators, 38 % by SF only, 6·3 % by sTfR only, while none had ID identified by TBI only. The mean Hb concentration was highest in those with no ID by any iron indicator (12·8 g/dl), lowest in those with ID by all indicators (11·6 g/dl), while 12·6 g/dl in those with ID by SF only (Fig. 1).

The prevalence of anaemia differed significantly between ethnic groups and was at least the double in all ethnic minority women compared with Western European women (26–40 % *v.* 14 %; *P* < 0·01–0·05) (Table 2). Regarding ID, the prevalence by TBI was twice as high in South Asians, Middle Easterners and Sub-Saharan Africans compared with Western Europeans. We found no significant ethnic differences for ID using SF <15 μg/l. East Asians had an overall better iron status by all indicators compared with the other ethnic groups in all four Hb concentration intervals, including those with anaemia (Hb concentration interval 8·0–11·9 g/dl) (Fig. 2).

After having excluded women with elevated CRP concentration >5 mg/l (9–26 % by ethnic groups), only minor changes in the mean and median values and in the prevalence of ID and anaemia across ethnic groups were observed (data not shown). Furthermore, using SF concentration <12 μg/l as the threshold for ID, the overall prevalence of ID declined from 39 to 29 % in the total sample, approaching the prevalence levels for sTfR and TBI (Supplementary Table S1).

### Associations between ethnicity and clinical factors with Hb, SF, sTfR and TBI

In unadjusted analyses, the Hb concentration was significantly lower in South Asians, Sub-Saharan Africans and East Asians compared with Western Europeans (Table 3). After adjusting for covariates, the ethnic differences were reduced and only South Asians had lower Hb concentration compared with Western European women. Gestational anaemia, other chronic illnesses/medication and postpartum haemorrhage were associated with lower Hb concentration, and higher age, self-reported intake of iron supplement in GW 28, and higher SEP in childhood were associated with higher Hb concentration.

Also for the ID indicators, ethnic differences were reduced after adjusting for covariates, as only South Asians had higher sTfR concentrations compared with Western Europeans (Table 3). Gestational ID and an ‘unhealthy’ dietary pattern were consistently associated with poorer iron status postpartum by all iron indicators. Postpartum haemorrhage was associated with higher OR of SF <15 μg/l and lower TBI concentration, and multiparous women and women with self-reported intake of iron supplement in the second trimester had better iron stores by all iron indicators.

### Associations between level of integration and clinical factors with Hb, SF, sTfR and TBI

Adjusting for SEP had minimal effect on the effect estimates (data not shown), and when exploring relations with variables reflecting the level of social integration, as alternatives to ethnicity, we found no or only weak associations with anaemia or the ID measures (Supplementary Table S2). In the ethnic minority sub-sample, we observed that the level of social integration could not explain the higher sTfR – and lower Hb concentrations found in South Asians compared with other ethnic minority groups (Supplementary Table S3).

## Discussion

To the best of our knowledge, this is one of very few studies from Europe to estimate the prevalence of postpartum anaemia and ID in a multi-ethnic population, and the only one comparing three indicators of ID, their relations and their relations to anaemia. One-fourth of the women had anaemia 14 weeks postpartum, two in five had ID by SF and about one in five had ID by sTfR or TBI. The mean Hb concentration was higher in those with ID by SF only (12·6 g/dl) than in those with ID by all indicators (11·6 g/dl). Women with ethnic origin outside Europe had a crude prevalence of anaemia and ID by sTfR and TBI that was about the double compared with European women. However, after adjusting for clinically relevant covariates, these ethnic differences mostly disappeared, with only South Asians having lower Hb concentration and higher sTfR concentration. The level of social integration into the Norwegian mainstream society did not explain these differences.

Different biomarkers are used to measure ID in clinical practice, SF being the most commonly used^(23)^. A Nordic study compared ID defined by SF <15 μg/l to bone marrow staining, and found SF to have a 75 % sensitivity and 98 % specificity^(24)^. The comparison of the different iron indicators revealed a larger dispersion for median SF concentration than mean sTfR concentration between the ethnic groups for the same Hb concentration interval, which could suggest that sTfR has less random variation and is a reliable iron indicator. Furthermore, Fig. 1 shows that the mean Hb concentration in those with ID by SF is only slightly influenced (mean Hb 0·2 g/dl lower compared with those with no ID by any marker), while it is significantly decreased in those with ID by sTfR and TBI (mean Hb 1·2 g/dl lower). This supports findings from others that SF covers an earlier stage of ID, called ‘depleted body iron stores’, while sTfR and TBI reflect a later stage of ID where the synthesis of Hb is affected^(11)^. This is also supported by a higher proportion of anaemia among those with ID by sTfR or TBI compared with those with ID by SF. To achieve an even higher concordance between ID assessed by SF and TBI, assuming TBI being a better predictor of ID, a lower threshold for ID by SF might perform better. This is supported by our findings that the prevalence of ID by SF <12 μg/l, also a widely used definition^(16)^, provided more comparable prevalence rates to those for sTfR and TBI (29 % *v.* 19 and 22 %, respectively). SF is an acute phase protein and the concentration is known to increase with infection and inflammatory processes, and can lead to an underestimate of ID^(17)^. sTfR and TBI are believed to better assess the severity of ID, as the sTfR is proportional to the cellular iron demand, but recent studies show that also these biomarkers may be affected by these processes, more specifically low-grade chronic inflammation that can result in an overestimate of ID^(11,12)^. We therefore ran the analyses in women without inflammatory response (CRP < 5), and found only minor changes in the mean/median concentration and prevalence rates of ID and anaemia across ethnic groups and conclude that inflammation could not explain the differences observed in our population. Therefore, we chose not to adjust SF, sTfR or TBI values for CRP to correct for inflammation, as suggested by some others^(11,12,17)^.

The crude total prevalence estimates for postpartum anaemia are in accordance with other studies from Europe^(1,25)^, the US^(26,27)^ and estimates published by the WHO^(2,28)^ when measured at least >8 weeks postpartum. Although ethnic minority background is recognised as a risk factor^(25,26,29–31)^, we did not find any studies from Europe reporting prevalence rates stratified by ethnic groups. The prevalence of postpartum anaemia in minority groups in our study was slightly lower than in the US^(32)^, but generally similar to those reported from their country of origin^(2)^, although prevalence rates reported from South Asian countries differ considerably (23–62 %)^(28)^.

We have only identified two studies reporting postpartum iron status^(26,33)^, and no studies comparing three different iron indicators or different ethnicities in postpartum women. The prevalence of postpartum ID defined by SF <12 μg/l in the NHANES study^(26)^ was about half of the prevalence in our study (13 % *v*. 29 %) and the mean SF concentration in postpartum women attending the special supplemental nutrition programme for women, infants and children (the WIC-programme) was higher than in our study (37 μg/l *v*. 23 μg/l)^(33)^. Of note, few women in our cohort used oral iron supplementation at postpartum. Studies among women in reproductive age, however, consistently indicate that ethnic minority and low SEP groups are at higher risk for the condition than the majority population^(6,34–38)^. In our study, East Asians had lower prevalence of ID (Fig. 2) and generally better iron status than the other ethnic minority groups. Although caution is needed in the interpretation of these findings due to the low number, East Asian women also had higher vitamin D concentrations compared with the other ethnic minority groups^(8)^, indicating that they may have a generally better nutritional status. Lastly, in line with others, we found that gestational anaemia and ID, an inadequate iron intake, and also postpartum haemorrhage were strongly associated with Hb concentration and poor postpartum iron status^(25–27,30,39,40)^.

Our study did not suggest that socioeconomic position or level of social integration played an important role in explaining ethnic differences in postpartum anaemia and ID. Women with South Asian origin had higher sTfR and lower Hb concentrations, both when compared with Western Europeans and with other ethnic minority groups, also after adjusting for covariates, including different measures of social integration, dietary pattern and life course SEP. We can, however, only speculate if this could be related to specific dietary factors among women with South Asian origin, such as Chapatti-based meals which contains a high level of phytates, a well-known inhibitor of iron absorption.

### Strength and limitations

The present study's major strength is its population-based cohort design with a high proportion of ethnic minorities, found to be fairly representative for the main ethnic groups of pregnant women living in Oslo, Norway. We present more robust data for anaemia than in our previous study from early pregnancy^(5)^, and were therefore able to compare the relations between three iron indicators and this clinical outcome. We have a broad, high-quality data set that enabled us to explore the relations between simultaneously measured Hb, and three indicators of ID and adjust for relevant covariates, and including socioeconomic conditions across the woman's life course, and we performed additional analyses to explore the impact of integration. There is, however, also limitations to report, including the possibility of heterogeneity within relatively broad ethnic groups. Furthermore, the number in some ethnic groups was low. We had some loss to follow-up at the postpartum visit, but we prioritised ethnic minority women for blood sampling. Low SF concentrations indicate ID, but different thresholds (<15 or <12 μg/ml) are used in the diagnosis of ID. To ease comparison with other studies, we primarily used the definition used by WHO when estimating ID^(16,23)^. We lack detailed information on iron intake, and postpartum haemorrhage was not measured exactly, but based on clinical judgement. We may also have underestimated the prevalence of haemoglobinopathy.

### Clinical implications

Hb measurements are often performed shortly after delivery, and only in women with postpartum haemorrhage or in women presenting symptoms of anaemia. In view of the clinical consequences of postpartum anaemia, a more active case-finding among high-risk women, such as most ethnic minority women, women with gestational anaemia and ID, and women with excessive postpartum bleeding seems needed – and could be implemented in clinical guidelines for later postpartum follow-up visit. This is also supported by the WHO target to reduce anaemia with 25 % by 2025^(41)^. Laboratory measurements are essential for a proper diagnosis of ID. Although more expensive, sTfR and TBI seem to assess the severity of ID better than SF. As it is considered clinically important to prevent the later stages of ID associated with IDA, our findings suggest that each iron indicator offers a slightly different interpretation of the physiological processes involved in the body's response to low iron stores. Further research is needed to disentangle the different stages and pathways in more detail.

## Conclusion

We present the first population-based study from Europe on postpartum anaemia and ID using three different iron indicators in a multi-ethnic sample of women. In total, 25 % of the women had postpartum anaemia measured 14 weeks after delivery. The prevalence of ID varied between 20 and 40 % by the different iron indicators. The current threshold values used to define ID by sTfR and TBI probably identified a more severe iron-deficient population compared with ID assessed by SF threshold values. Gestational anaemia or ID, insufficient iron intake in pregnancy and postpartum haemorrhage were independent risk factors of postpartum anaemia and ID, but women with South Asian origin had more anaemia and ID by sTfR, even when adjusting for covariates. To improve women's postpartum health status, clearer recommendation about measuring Hb and iron status in women at risk should be implemented in clinical guidelines in Norway and internationally.

## Supplementary material

For supplementary material accompanying this paper visit https://doi.org/10.1017/jns.2022.45. click here to view supplementary material
