# Supplementary material for: Prevalence of postpartum anaemia and iron deficiency by serum ferritin, soluble transferrin receptor and total body iron, and associations with ethnicity and clinical factors: a Norwegian population-based cohort study
Source: J Nutr Sci. 2022 Jun 13;11:e46. doi: 10.1017/jns.2022.45 (PMC9201879; doi:10.1017/jns.2022.45)
Supplement: Supplementary file 1 [file jnssup.zip › S2048679022000453sup001.docx]

Supplementary material:

1. Supplemental Table 1
2. Supplemental Table 2
3. Supplemental Table 3
4. Supplemental Figure 1

Supplemental Table 1

Title: Sensitivity analyses of the prevalence of iron deficiency by serum ferritin (SF) <15 µg/L and serum ferritin (SF) <12 µg/L 14 weeks postpartum in the STORK-Groruddalen study, and the relative change of prevalence of iron deficiency when the lower thresholds for serum ferritin is chosen ^1^

|  |  | SF <15 µg/L | SF <12 µg/L | Relative change |
| --- | --- | --- | --- | --- |
|  | n | % | % | % |
| Total sample | 573 | 39 | 29 | -26 |
| Ethnic group |  |  |  |  |
| Western Europe | 217 | 35 | 24 | -31 |
| South Asia | 157 | 44 | 35 | -20 |
| Middle East | 94 | 39 | 30 | -23 |
| Sub-Saharan Africa | 38 | 50 | 40 | -20 |
| East Asia | 33 | 36 | 24 | -33 |
| Eastern Europe | 34 | 32 | 29 | -9 |

^1^ The STORK-Groruddalen multi-ethnic pregnancy cohort from Oslo, Norway, 2008 – 2010.

Supplemental Table 2.

Title: Multivariable regression analyses exploring the potential effect of participants’ level of integration as an alternative to ethnicity. Logistic regression analysis of SF <15 µg/L, and linear regression analyses of sTfR, TBI, and Hb concentration 14 weeks postpartum in the STORK-Groruddalen study ^1^

|  |  |  |  |  |  |  |  |  |
| --- | --- | --- | --- | --- | --- | --- | --- | --- |
|  | SF <15 µg/dL | | sTfR, *mg/L* | | TBI, *mg/kg* | | Hb, *g/dL* | |
|  | adj OR | 95% CI | adj β | 95% CI | adj β | 95% CI | adj β | 95% CI |
|  | R^2^ = 0.13 | | R^2^ = 0.12 | | R^2^ = 0.15 | | R^2^ = 0.12 | |
|  |  |  |  |  |  |  |  |  |
| Low social integration (high = reference) ^2^ | 1.5 | 1.0, 2.2 | 0.02 | -0.3, 0.3 | -0.7 | -1.4, -0.1 * | -0.1 | -0.3, 0.1 |
| Postpartum week |  |  | -0.1 | -0.1, -0.02 ** |  |  |  |  |
| Age, *per 5 year* |  |  |  |  | 0.5 | 0.2, 0.9 * | 0.1 | 0.02, 0.2 * |
| Multiparous (primiparous = reference) | 0.5 | 0.4, 0.8 ** | -0.3 | -0.6, -0.01 * | 0.7 | -0.1, 1.4 | -0.2 | -0.4, 0.0 * |
| Early life socioeconomic position ^3^ |  |  |  |  |  |  | 0.2 | 0.1, 0.3 ** |
| Gestational ID or anaemia (no = ref) ^4^ | 1.5 | 0.9, 2.5 | 1.9 | 1.3, 2.6 ** | -2.7 | -3.7, -0.6 ** | -1.0 | -1.4, -0.6 ** |
| Iron supplementation use in GW 28 (no = reference) ^5^ | 0.5 | 0.3, 0.8 ** | -0.5 | -0.8, -0.2 ** | 1.2 | 0.6, 1.8 ** | 0.1 | -0.03, 0.3 |
| Dietary pattern (healthy = reference) ^6^ | 1.6 | 1.1, 2.5 * | 0.7 | 0.4, 1.1 ** | -1.7 | -3.7, -1.8 ** | -0.1 | -1.4, -0.7 |
| Chronic illness/medication associated with normochromic anaemia (no = reference) ^7^ |  |  |  |  |  |  | -0.8 | -1.4, -0.3 ** |
| Chronic illness/medication associated with hypochromic anaemia (no = reference) ^8^ |  |  |  |  |  |  | -0.05 | -0.3, 0.2 |
| Postpartum haemorrhage (<500 mL = reference) ^9^ | 3.1 | 1.4, 6.7 ** | 0.2 (-0.4, 0.8) |  | -1.9 | -3.1, -0.6 | -0.3 | -0.7, 0.003 * |

Adj, adjusted; GW, gestational week; Hb, haemoglobin; ID, iron deficiency; SEP, socioeconomic position; SF, serum ferritin; sTfR, soluble transferrin receptor; TBI; total body iron.

^1^ The STORK-Groruddalen multi-ethnic pregnancy cohort from Oslo, Norway, 2008 – 2010. Only adjusted values presented.

^2^ Variable derived from a principal components analysis of predefined markers reflecting integration such as language skills, time of residence, social interaction with ethnic Norwegians and use of Norwegian media, with a higher score reflect higher social integration. “Low social integration” represents participants belonging to the 40% with the lowest scores.

^3^ Variable derived from a principal components analysis of three childhood sociodemographic variables representing maternal SEP at age 10 years, with a higher score reflecting higher socioeconomic position.

^4^ Gestational iron deficiency by 1) SF <15 µg/L; 2) sTfR >4.4 mg/L, or 3) TBI <0 mg/kg; and gestational anaemia by trimester-specific haemoglobin < 10.5 or 11.0 g/dL, analysed in mean gestational week 15.1.

^5^ Selfreported intake of iron supplements during the past two weeks in GW 28.

^6^ Data from food frequency questionnaires collected in GW 28; four clusters were extracted using the Ward’s method. Clusters were referred to as “a healthier dietary pattern” vs. three “less healthy dietary patterns”; here dichotomized into “healthy” and “unhealthy” dietary pattern.

^7^ Self-reported chronic illness or medication associated with normochromic anaemia (i.e. kidney or rheumatic disease, use of carbamazepine or infliximab).

^8^ Self-reported chronic illness or medication associated with ID and hypochromic anaemia (i.e. gastrointestinal disease or Copper intrauterine device use before conception).

^9^ Excessive blood loss (≥500 mL) after delivery.

* *P*<0.05, ** *P*<0.01.

Supplemental Table 3

Title: Logistic regression analysis of serum ferritin <15 µg/L, and linear regression analyses of soluble transferrin receptor, total body iron, and haemoglobin concentration 14 weeks postpartum in a sub-sample of non-Western women in the STORK-Groruddalen study^1^.

|  |  |  | SF <15 µg/dL | |  |  | sTfR, *mg/L* | |  |  | TBI, *mg/kg* | |  |  | Hb, *g/dL* | |  |
| --- | --- | --- | --- | --- | --- | --- | --- | --- | --- | --- | --- | --- | --- | --- | --- | --- | --- |
|  |  | OR | 95% CI | adj OR | 95% CI | β | 95% CI | adj β | 95% CI | β | 95% CI | adj β | 95% CI | β | 95% CI | adj β | 95% CI |
|  |  |  |  | R^2^ = 0.12 | |  |  | R^2^ = 0.12 | |  |  | R^2^ = 0.16 | |  |  | R^2^ = 0.15 | |
|  |  |  |  |  |  |  |  |  |  |  |  |  |  |  |  |  |  |
| Non-Western ethnic origin except South Asians ^1^ | |  |  |  |  |  |  |  |  |  |  |  |  |  |  |  |  |
| South Asia | | 1.2 | 0.8, 1.8 | 1.2 | 0.7, 1.9 | 0.5 | 0.1, 1.0 ** | 0.5 | -0.9, -0.1 * | -0.7 | -1.5, -0.1 | -0.5 | -0.3, -1.4 | 0.5 | -0.7, 0.3 ** | 0.5 | -0.7, 0.3 ** |
| Postpartum week | | 1.0 | 0.9, 1.0 |  |  | -0.1 | -0.1, -0.01 * | -0.1 | -0.2, -0.001 | 0.1 | -0.04, 0.2 |  |  | 0.02 | -0.01, 0.05 |  |  |
| Age, *per 5 year* | | 0.8 | 0.7, 1.0 * |  |  | -0.2 | -0.4, -0.1 ** |  |  | 0.7 | 0.4, 1.0 ** | 0.5 | 0.1, 1.0 * | 0.1 | -0.01, 0.02 | 0.1 | -0.03, 0.2 |
| Multiparous (primiparous = reference) | | 0.6 | 0.4, 0.8 ** | 0.6 | 0.4, 0.9 * | -0.2 | -0.5, 0.1 | -0.4 | 0.8, -0.03 | -0.1 | -0.4, 0.2 | 1.0 | 0.1, 1.9* | -0.03 | -0.1, 0.1 | -0.2 | -0.4, 0.1 |
| Pre-pregnant Body Mass Index, *per 5 kg/m^2^* | | 1.0 | 0.8, 1.2 |  |  | 0.2 | 0.03, 0.3 * |  |  | -0,1 | -0.4, 0.4 |  |  | 0.03 | -0.1, 0.1 |  |  |
| Adult socioeconomic position ^2^ | | 0.8 | 0.7, 0.9 * |  |  | -0.3 | -0.4, 0.1 ** |  |  | 0.6 | 0.3, 0.9 ** |  |  | 0.2 | 0.1, 0.2 ** |  |  |
| Social integration ^3^ | | 1.0 | 0.9, 1.2 | 1.2 | 1.0, 1.5 | -0.2 | -0.3, -0.01 * | 0.03 | -0.2, 0.2 | 0.2 | -0.2, 0.5 | -0.2 | -0.6, 0.1 | 0.1 | 0.03, 0.2 ** | 0.05 | -0.05, 0.1 |
| Early life socioeconomic position ^4^ | | 0.9 | 0.7, 1.0 |  |  | -0.2 | -0.4, -0.1 ** |  |  | 0.3 | 0.01, 0.6 * |  |  | 0.2 | 0.1, 0.3 ** | 0.1 | 0.01, 0.3 * |
| Gestational ID or anaemia (no = reference) ^5^ | | 1.3 | 0.9, 1.8 | 1.5 | 0.9, 2.4 | 2.0 | 1.4, 2.6 ** | 1.8 | 1.0, 2.5 ** | -2.5 | -3.4, -1.5 ** | -2.6 | -3.8, -1.3 ** | -0.9 | -1.3, -0.6 ** | -1.0 | -1.4, -0.7 ** |
| Iron supplementation use in GW 28 (no = reference) ^6^ | | 0.6 | 0.4, 0.9 * | 0.6 | 0.4, 1.0 * | -0.3 | -0.6, -0.004 * | -0.7 | -1.2, -0.3 ** | 0.8 | 0.1, 1.4 * | 1.5 | 0.7, 2.3 ** | -0.002 | -0.2, 0.2 | 0.2 | -0.04, 0.4 |
| Dietary pattern (healthy = reference) ^7^ | | 2.2 | 1.5, 3.3 ** | 5.2 | 2.1, 12.7 ** | 0.6 | 0.3, 1.0 ** | 0.9 | 0.3, 1.6 ** | -1.5 | -2.2, -0.8 ** | -2.7 | -2.1, -0.5 ** | -0.2 | -0.4, -0.1 ** | -0.1 | -0.4, 0.2 |
| Chronic illness/medication associated with normochromic anaemia (no = reference) ^8^ | | 1.5 | 0.5, 4.4 |  |  | 0.8 | -0.1, 1.7 |  |  | -0.1 | -2.0, 1.9 |  |  | -0.8 | -1.3, -0.2 ** | -1.0 | -1.7, -0.4 ** |
| Chronic illness/medication associated with hypochromic anaemia (no = reference) ^9^ | | 0.7 | 0.4, 1.3 |  |  | -0.2 | -0.7, 0.3 |  |  | 0.4 | -0.7, 1.4 |  |  | -0.1 | -0.3, 0.2 | 0.1 | -0.3, 0.5 |
| Operative delivery (no = reference)  ^10^ | | 1.3 | 0.9, 1.9 |  |  | -0.03 | -0.3, 0.3 |  |  | -0.5 | -1.2, 0.1 |  |  | 0.01 | -0.2, 0.2 |  |  |
| Postpartum haemorrhage (<500 mL = reference) | | 2.9 | 1.4, 6.0 ** | 4.0 | 1.3, 12.8 * | 0.3 | -0.3, 0.9 | 0.3 | -0.6, 1.3 | -1.6 | -2.9, -0.3 * | -2.1 | -3.9, -0.2 * | -0.2 | -0.5, 0.2 | -0.4 | -0.9, -0.05 |
| Birth complications (no = reference) ^11^ | | 1.4 | 0.9, 2.2 |  |  | 0.07 | -0.3, 0.5 |  |  | -0.8 | -1.6, -0.04 * |  |  | -0.1 | -0.3, 0.2 |  |  |

Adj, adjusted, GW, gestational week; Hb, haemoglobin; ID, iron deficiency; SEP, socioeconomic position; SF, serum ferritin; sTfR, soluble transferrin receptor; TBI; total body iron.

^1^ The STORK-Groruddalen multi-ethnic pregnancy cohort from Oslo, Norway, 2008 – 2010. Multivariable regression analyses exploring the potential effect of participants’ socioeconomic position and integration. Western Europeans were excluded from this model. Non-Western women were stratified into South Asian and other ethnic minority origin.

^2^ Variable derived from a principal components analysis of predefined individual and household markers of socioeconomic position (SEP), with a higher score reflect higher SEP.

^3^ Variable derived from a principal components analysis of predefined markers reflecting integration such as language skills, time of residence, social interaction with ethnic Norwegians and use of Norwegian media, with a higher score reflect higher social integration.

^4^ Variable derived from a principal components analysis of three childhood sociodemographic variables representing maternal SEP at age 10 years, with a higher score reflecting higher SEP.

^5^ Gestational iron deficiency by 1) SF <15 µg/L; 2) sTfR >4.4 mg/L, or 3) TBI <0 mg/kg; and gestational anaemia by trimester-specific haemoglobin < 10.5 or 11.0 g/dL, analysed in mean gestational week 15.1.

^6^ Self-reported intake of iron supplements during the past two weeks at all three study visits dichotomized into “yes”, covering daily or intermittent iron supplements, and “no”.

^7^ Data from food frequency questionnaires collected in GW 28; four clusters were extracted using the Ward’s method. Clusters were referred to as “a healthier dietary pattern” vs. three “less healthy dietary patterns”; here dichotomized into “healthy” and “unhealthy” dietary pattern.

^8^ Self-reported chronic illness or medication associated with normochromic anaemia (i.e. kidney or rheumatic disease, use of carbamazepine or infliximab).

^9^ Self-reported chronic illness or medication associated with ID and hypochromic anaemia (i.e. gastrointestinal disease or Copper intrauterine device use before conception).

^10^ Operative delivery: Caesarean section (elective and emergency) or assisted vaginal delivery (forceps or vacuum). Normal vaginal delivery reference.

^11^ A composite variable created by combining following four birth complication; episiotomy, third- and fourth degree perineal tear, obstructed labor and manual removal of placenta.

* *P*<0.05, ** *P*<0.01.

Supplemental Figure 1

Title: Flow chart of study participants in the STORK-Groruddalen multi-ethnic pregnancy cohort from Oslo, Norway, 2008 – 2010.

Hb, haemoglobin; SF, serum ferritin; sTfR, soluble transferrin receptor; TBI; total body.

^1^ Information, material and questionnaires were translated into Arabic, English, Sorani, Somali, Tamil, Turkish, Urdu and Vietnamese and quality checked by bilingual health professionals.

^2^ Abortion, stillbirth, preterm birth, twin birth, sick at the postpartum visit (n=30). Did not attend due to logistic/unknown reasons (n=118).

^3^ At the postpartum visit, due to resource limitations (including sick leave among study staff at one study site), women with ethnic minority background were prioritized for fasting blood samples. We lack data on SF concentration for seventy-one women, primarily ethnic Norwegians.

^4^ Missing value of Hb (n=4), sTfR (n=5) and TBI (n=5).
