# Supplementary figures and images for: Prevalence of postpartum anaemia and iron deficiency by serum ferritin, soluble transferrin receptor and total body iron, and associations with ethnicity and clinical factors: a Norwegian population-based cohort study
Source: J Nutr Sci. 2022 Jun 13;11:e46. doi: 10.1017/jns.2022.45 (PMC9201879; doi:10.1017/jns.2022.45)

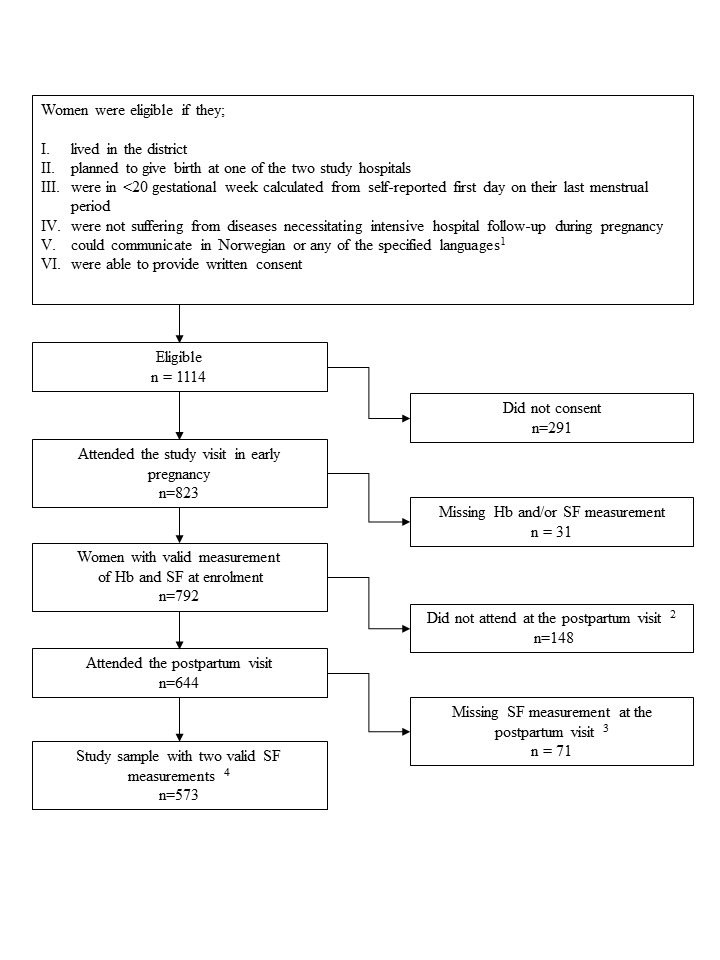

Supplement: Supplementary file 1 [file jnssup.zip › S2048679022000453sup002.tif]
